# The Impact of Dietary Guidance During Cancer Treatment on Quality of Life

**DOI:** 10.3390/nu18132097

**Published:** 2026-06-26

**Authors:** Vera Ósk Guðjónsdóttir, Lára Kristjánsdóttir, Kristjana Sigurðardóttir, Jóhanna Eyrún Torfadóttir

**Affiliations:** 1 Centre of Public Health Sciences, School of Health Sciences, University of Iceland, 102 Reykjavík, Iceland; vog4@hi.is; 2 Icelandic Cancer Registry and Research Center at the Icelandic Cancer Society, 105 Reykjavík, Iceland; lara@krabb.is (L.K.);

**Keywords:** cancer treatment, nutritional guidance, quality of life, survivorship care

## Abstract

**Background/Objectives**: We examined whether guidance on dietary habits or nutrition-related problems from healthcare professionals during cancer treatment was associated with quality of life, after treatment. **Methods**: Cross-sectional data were drawn from the Icelandic Compass study, conducted in 2020–2021 among adults diagnosed with cancer in 2015–2019. The analysis included participants who had completed treatment for breast cancer (n = 341), prostate cancer (n = 137), or colorectal cancer (n = 132), for a total sample of 610 participants. Quality of life (QL) was assessed using the EORTC QLQ-C30 global health status/quality of life scale. Associations were examined using regression models adjusted for age, marital status, education, number of cancer treatments, stage at diagnosis, body mass index, tobacco and alcohol use, and comorbidities. **Results**: Overall, 26% of participants reported receiving sufficient guidance on general dietary habits during treatment and 19% on nutrition-related problems. On average, three years had passed since diagnosis. Among all participants, guidance on general dietary habits was associated with higher QL scores (β = 5.6; 95% CI: 0.8 to 10.5), as was guidance on nutrition-related problems (β = 5.7; 95% CI: 0.3 to 11.1). In subgroup analyses, statistically significant associations were observed among prostate cancer survivors for both dietary guidance (β = 12.4) and guidance on nutrition-related problems (β = 14.0), and among breast cancer survivors for guidance on nutrition-related problems (β = 8.4). **Conclusions**: Patient-reported sufficient discussions about dietary habits or nutrition-related problems during treatment were associated with slightly higher post-treatment QL scores.

## 1. Introduction

Cancer continues to exert a major toll on health worldwide [1]. Advances in detection and treatment have increased survival, leading to a growing population living with ongoing challenges related to cancer and its therapies and possible side effects, making interventions that address quality of life increasingly essential [2].

In 2022, an estimated 53.5 million people were living within five years of a cancer diagnosis, while approximately 20 million new cancer cases and 9.7 million cancer-related deaths were recorded worldwide, yet access to comprehensive cancer care remains uneven [1]. This disparity is mostly due to medical advances being more available in high-income countries. High-income countries have larger older populations, who are also at a higher risk for cancer. Higher life expectancy and higher cancer survival rates mean high-income countries have more people who live longer with cancer and often require ongoing management for treatment-related disabilities and chronic health issues [3].

The overall burden of cancer is expected to increase in the coming decades, with projections of up to 35 million diagnosed cases by 2050 [1]. As survival improves, many patients face treatment and disease-related complications such as malnutrition and metabolic disorders that substantially reduce their quality of life. Some estimates indicate that 40–80% of cancer patients experience malnutrition at some point during their illness [3,4]. Malnutrition and related metabolic diseases worsen treatment tolerance, increase complications, and contribute to functional decline. Addressing these complications, especially through targeted dietary counseling, may be vital to ensuring quality of life for survivors.

In Iceland, cancer incidence and survival patterns broadly reflect high-income country trends, with breast, prostate, and colorectal cancers among the most frequent cancer diagnoses [5]. National policy in Iceland recognizes the right of cancer patients to individual support, including nutritional assessment where appropriate [6]. Despite these recommendations, service provision varies. An Icelandic study found that only about 19% of participants diagnosed with cancer between 2015 and 2019 reported being assessed for the need for nutritional counseling, including 24% of men and 16% of women [7]. This suggests that limited implementation of nutritional screening and dietary counseling may reduce opportunities to support quality of life among cancer patients in Iceland. Cancer and its treatment can significantly affect patients’ cognitive and physical abilities, with up to 35% of cancer survivors experiencing long-term post-treatment cognitive impairments [8]. Physical function may also be impaired through increased fatigue, muscle loss, and reduced mobility [9]. Nutrition and cognition may influence each other, as malnutrition can contribute to declines in cognitive function, while cognitive deficits may reduce appetite and food intake and make dietary guidance harder to follow [10]. Home-delivered meals have been associated with improved cognitive function and well-being among older adults [11].

Qin et al. (2023) [12] reported that physical function varied by cancer type during active disease, with patients diagnosed with breast, gynecological, and colorectal cancers showing relatively better physical function than those with other cancer types. However, among individuals with non-active cancer, cancer type was no longer a major determinant of physical function; instead, comorbidities and general risk factors appeared to play a greater role [12]. Importantly, functional impairments may persist long-term. Another study showed that women with a history of cancer had lower physical function than age-matched controls even years after diagnosis and treatment [13].

Several studies suggest that nutritional counseling may improve global health status, quality of life (QL), and nutritional status among cancer patients. In a randomized controlled trial among colorectal cancer patients undergoing radiotherapy, Ravasco et al. found that individualized dietary counseling improved nutritional intake, nutritional status, and QL, with benefits persisting three months after treatment [14]. Similarly, systematic reviews suggests that individualized dietary counseling may help maintain body weight, reduce malnutrition, mitigate chemotherapy-induced gastrointestinal toxicity, improve treatment tolerance, and support self-management and QL [15,16].

According to the European Cancer Organisation, comorbidities are common among cancer patients, with the proportion reporting at least one comorbid condition reaching up to 90% depending on age and type of cancer, which can affect treatment decisions [17]. In an Icelandic study among individuals diagnosed with cancer, participants had a mean age of 59 years, and 58% reported at least one comorbidity [7].

The effects of malnutrition extend beyond physical decline. Poor nutritional status is associated with poorer treatment outcomes, reduced survival, and worse psychological well-being [18]. Malnutrition and cachexia may also reduce treatment tolerance, increase complications, prolong hospital stays, and lower survival [3].

Clinical guidelines recommend routine nutritional screening and early, individualized interventions for patients at risk of malnutrition [3]. Nutritional counseling may help relieve symptoms such as nausea and appetite loss, stabilize body weight, support physical function and psychological well-being, and improve QL [2]. However, whether nutrition-related guidance during cancer treatment is associated with better QL scores after treatment remains unclear.

This study aimed to examine the association between receiving nutritional guidance during cancer treatment and self-reported quality of life after treatment.

## 2. Materials and Methods

### 2.1. Study Design and Participants

This study uses data from the Compass study, a nationwide cross-sectional study conducted by the Icelandic Cancer Society (ICS) from 2020 to 2021. The Compass was modeled after a Danish survey called “the Barometer survey” [19] and was adapted to Icelandic conditions, with added questions regarding lifestyle and demographic factors. The Barometer survey comprises questions regarding patients’ experiences of the diagnostic process, treatment, and rehabilitation, as well as questions on aspects that define the quality of life (QL) of cancer patients or survivors. The Icelandic Cancer Society’s study The Compass invited individuals diagnosed with cancer between 2015 and 2019, totaling 4575 individuals [7]. Individuals with carcinoma in situ of the breast were not offered participation. Of those invited, 340 individuals were diagnosed with non-melanoma skin cancer and were therefore not sent participation reminders because this diagnosis may not always be perceived as cancer. Of the 4575 eligible participants, 1831 enrolled in the study, yielding a participation rate of 40%. Data were collected from 8 June 2020, to 1 May 2021, and 1672 participants completed the online questionnaire.

**Figure 1:**
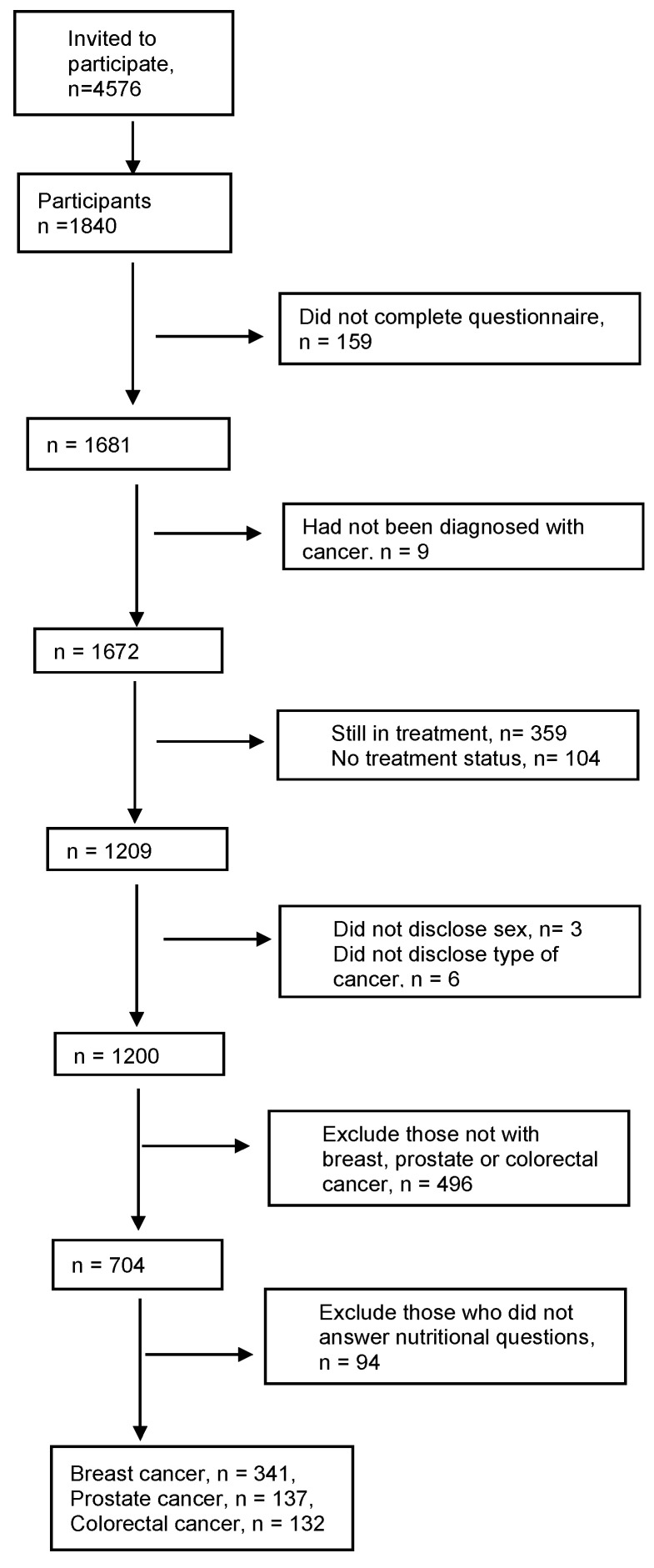
Diagram of participant selection, including reasons for exclusion and missing data.

The present analysis was restricted to participants who had completed cancer treatment, responded to questions on dietary guidance during treatment, and had been diagnosed with one of the three most common cancer types in the study population: breast, prostate, or colorectal cancer (n = 610). The full exclusion criteria are presented in Figure 1.

Among the participants, 56% had breast cancer (n = 341), 22% had prostate cancer (n = 137), and 22% had colorectal cancer (n = 132), as can be seen in Figure 1. Due to the limited number of males with breast cancer, they were excluded from the breast cancer group.

### 2.2. Exposure Variable

The survey included questions asking participants whether a healthcare professional discussed their dietary habits or nutritional problems during cancer treatment. The question was “Has the healthcare staff at a hospital discussed the following subject with you?” The answer options are shown in Table 1 Responses were categorized into three groups—yes (received guidance sufficiently), to some extent/slightly and no (did not receive guidance). The group comprising participants who responded “to some extent” or “only slightly” was not examined separately in the analyses on quality of life.

As outlined above, responses were categorized into three groups. Participants who responded “yes, sufficiently” were classified as “yes,” while those who responded “yes, to some extent” or “only slightly” were combined into one group and finally those who responded, “to no extent” were classified as “no.” Participants who answered “do not remember” or “not applicable” were excluded.

### 2.3. Outcome Variables

Health-related quality of life and functioning were assessed using the EORTC QLQ-C30, a widely used 30-item questionnaire developed for cancer populations [20] at study entry. The EORTC QLQ-C30 includes five functional scales, three symptom scales, six single-item measures, and one global health status/quality of life scale [21]. In the present study, the outcome variables were global health status/QL scale, physical functioning, and cognitive functioning.

The global health status/QL scale consists of two items assessing overall health and overall quality of life during the past week. Responses are given on a 7-point scale ranging from 1 = “Very poor” to 7 = “Excellent.” Physical functioning was assessed using five items, and cognitive functioning using two items, both scored on a 4-point scale ranging from 1 = “Not at all” to 4 = “Very much.”

All scores were linearly transformed to a 0–100 scale according to the EORTC scoring manual, with higher scores indicating better QL or functioning. Participants who answered “cannot answer/do not know” were excluded from the relevant analyses. Missing values were imputed using the mean of the completed items within a scale, provided that at least half of the items in that scale had been answered, in line with EORTC recommendations [21].

Clinically relevant thresholds were used to classify participants as having high or poor QL and functioning. For physical and cognitive functioning, thresholds were based on Giesinger et al. (2020) [22], with scores of 83 or higher indicating high physical functioning and scores of 75 or higher indicating high cognitive functioning. Scores below these thresholds were classified as poor functioning [22]. For global health status/QL, a cutoff of 50 was used, based on Diouf et al. (2015) [23], due to variation in proposed thresholds for clinical importance. Accordingly, QL scores of 50 or higher were classified as high, while scores below 50 were classified as poor [23].

### 2.4. Covariates

We retrieved information on potential confounders collected via the questionnaire at study entry in the Compass study. Covariates found there were age (continuous), sex (male; female; other), marital status (married or cohabitating; in a relationship; single; widow/er; cannot answer/do not know), number of children under 18 years old (continuous), educational level (primary; upper secondary; trade; university), alcohol intake (does not drink; once a month or less; two to three times a month; once a week; twice a week, three to six times a week; daily; cannot answer/do not know), smoking history (no, never smoked; no but used to smoke; yes, but not daily; yes, daily; cannot answer/do not know), cancer stage (1; 2; 3 or 4), BMI (continuous), number of treatments (none, one, two, three or more) and comorbidities (yes = one or more of these diseases: diabetes; pulmonary disease; mental health problems; cardiovascular disease; musculoskeletal disorders; neurological disease; metabolic disease; another disease or health condition,; no; cannot answer/do not know).

Information on the stage of cancer at diagnosis was obtained from the cancer registry for about 80% of the study participants.

### 2.5. Statistical Analysis

Linear regression was used to examine the associations between the two exposure variables, reported discussions with healthcare professionals about general dietary habits and nutrition-related problems, and three outcomes: Global quality of life (QL) scale, physical functioning (PF), and cognitive functioning (CF). Analyses were conducted separately for breast, prostate, and colorectal cancer survivors, as well as for all three cancer groups combined. Results are presented as adjusted mean differences (β), 95% confidence intervals (CI), *p*-values, and sample sizes.

The regression coefficients represent the adjusted mean difference in each outcome associated with reported dietary or nutrition-related discussions during treatment, compared with no such discussion. Models were adjusted for age, number of children under 18 years, educational level, marital status, number of cancer treatments, stage at diagnosis, comorbidities, body mass index (BMI), alcohol intake, and smoking history. In analyses restricted to colorectal cancer survivors, models were additionally adjusted for sex. Stage at cancer diagnosis could not be included in the models.

Statistical significance was assessed using *p*-values, with *p* < 0.05 considered statistically significant.

## 3. Results

### 3.1. Study Characteristics

In total, there were 610 participants in this study who comprised all those diagnosed with either breast, n = 341 (mean age at diagnosis 57.5 ± 10.5), prostate, n = 137 (mean age at diagnosis 65.9 ± 7.0), or colorectal, n = 132, cancer (mean age at diagnosis 62.4 ± 9.1). Among them, 530 answered the question about nutrition-related problems. Most participants in the Compass study were female, or 60%, and the gender distribution was almost identical in this study, with 59% of participants being female. In total, 26% discussed sufficiently dietary habits with healthcare professionals during cancer treatment, and 19% discussed sufficiently nutritional problems. On average, participants entered the study approximately three years after cancer diagnosis and the mean QL score at study entry was 72.7 points ± 20.0. The mean time since diagnosis was similar in both groups: 3.1 years among those who received dietary guidance and 3.0 years among those who did not. 

### 3.2. Breast Cancer

The breast cancer group included 341 participants and 300 of them answered the question about nutrition-related problems. On average, participants with breast cancer were the youngest cancer group in the study. The mean time from diagnosis to study participation was 3.1 ± 1.5 years, and the mean QL score at study entry was 70.6 points ± 20.4.

Information on the stage of cancer at diagnosis was available for 78% of the women with breast cancer, and about half of them (49%) had stage I at diagnosis, 37% had stage II, and 14% had stage III or IV at diagnosis. Most women with breast cancer (95%) reported receiving two or more types of cancer treatments. About 50% of the women also reported having one or more comorbidities before the diagnosis.

During cancer treatment, 23% reported receiving sufficient guidance on dietary habits, and 18% reported receiving sufficient guidance on nutritional problems (Table 2). Participants who reported sufficient discussions about general dietary habits or nutrition-related problems tended to be younger, were more likely to have children under 18 years at home, had slightly higher BMI, and were more often diagnosed with higher-stage cancer. They were also less likely to be current smokers and, particularly for discussions about general dietary habits, appeared to have lower educational attainment. Participants reporting sufficient discussions had a lower prevalence of poor quality of life. For nausea/vomiting and appetite loss during the past seven days, symptom scores were lower among those reporting sufficient discussions than among those reporting discussions to some extent or slightly, but not consistently lower than among those reporting no discussions.

### 3.3. Prostate Cancer

Table 3 presents the characteristics of the prostate cancer group, which included 137 participants and 117 of them answered the question about nutrition-related problems. The mean time from diagnosis to study participation was 3.2 ± 1.6 years, and the mean QL score at study entry was 77.0 points ± 18.1. Information about the stage of cancer at diagnosis was available for 80% of men with prostate cancer, and about half of them (49%) had stage I at diagnosis, 23% had stage II, and 28% had stage III or IV at diagnosis. A modest majority of men with prostate cancer (60%) reported receiving one or no cancer treatments. About 54% of the men with prostate cancer also reported having one or more comorbidities before the diagnosis. During cancer treatment, 21% reported receiving sufficient guidance on dietary habits, and 16% received sufficient guidance on nutritional problems.

Among participants with prostate cancer, those who reported sufficient discussions about general dietary habits had slightly higher BMI and appeared to have lower educational attainment than those reporting no such discussions. They also reported lower alcohol consumption, with none reporting alcohol intake three or more times per week. Participants reporting sufficient discussions about general dietary habits were more often diagnosed with higher-stage cancer, and poor quality of life and poor cognitive functioning were less common in this group than among those reporting no discussions. Constipation during the past seven days also appeared to be lower among those reporting sufficient discussions during cancer treatment.

For discussions about nutrition-related problems, participants who reported sufficient discussions also had slightly higher BMI, lower educational attainment, lower alcohol consumption, and fewer comorbidities than those reporting no such discussions. They appeared to be somewhat younger than those reporting no discussions, although the youngest group was those reporting discussions to some extent or slightly. Poor quality of life and poor cognitive functioning were also less common among those reporting sufficient discussions about nutrition-related problems during cancer treatment, and constipation during the past seven days appeared to be lower in this group.

### 3.4. Colorectal Cancer

There were 132 participants with colorectal cancer included in the study and 113 of them answered the question about nutrition-related problems (Table 4). The mean time from diagnosis to study participation was 2.9 ± 1.6 years, and the mean QL score at study entry was 73.2 points ± 20.4. Most participants were men (62%). Information on cancer stage at diagnosis was available for 86% of participants. Among those with available stage information, 25% had stage I disease, 24% had stage II disease, and 51% had stage III or IV disease.

A modest majority of participants (65%) had received two or more types of cancer treatment, and 61% reported having one or more comorbidities before diagnosis. During cancer treatment, 38% reported receiving sufficient guidance on dietary habits, and 27% reported receiving sufficient guidance on nutritional problems.

Among participants with colorectal cancer, gastrointestinal symptoms appeared to be more pronounced during the past seven days than in the other cancer groups, approximately three years after diagnosis. This was particularly evident for diarrhea and constipation.

For discussions about general dietary habits, participants who reported sufficient discussions tended to be somewhat younger, were more often married or in a relationship, more often had a university degree, and more often reported having one or more comorbidities than those reporting no such discussions. They were also more often diagnosed with higher-stage cancer. Poor physical functioning was less common among participants reporting sufficient discussions about general dietary habits than among those reporting no discussions.

For discussions about nutrition-related problems, participants who reported sufficient discussions were also more often diagnosed with higher-stage cancer. They appeared slightly more likely to have received two or more types of cancer treatment than those reporting no discussions, although this difference was small and the highest proportion was observed among those reporting discussions to some extent or slightly.

### 3.5. General Dietary Habits and Quality of Life

Table 5 shows that, in the combined sample across all three cancer types, reported sufficient discussions about general dietary habits were associated with higher QL scores after treatment (β = 5.6 points; 95% CI: 0.8 to 10.5; *p* = 0.02) in a fully adjusted model (N = 443). In the model without adjusting for cancer stage at diagnosis (N = 547), participants who reported sufficient discussions had QL scores that were, on average, 7.6 points higher than those who did not (95% CI: 1.7 to 13.6; *p* = 0.01).

In analyses stratified by cancer type, a statistically significant association was observed only among prostate cancer survivors, where those who reported sufficient discussions had higher QL scores than those who did not (β = 12.4; 95% CI: 2.5 to 22.2; *p* = 0.01). Among breast cancer survivors, the estimate suggested a possible positive association, although it did not reach statistical significance (β = 6.4; 95% CI: −0.3 to +13.2; *p* = 0.07).

No statistically significant associations were observed between sufficient guidance on dietary habits and physical functioning (PF) or cognitive functioning (CF) in the combined sample. The adjusted mean differences were β = 4.1 for PF (95% CI: −1.0 to +9.1; *p* = 0.11) and β = 3.0 for CF (95% CI: −2.5 to +8.5; *p* = 0.28). Similarly, no statistically significant associations were observed in the cancer-specific subgroup analyses, as shown in Table 5, although in the model, without adjusting for stage at diagnosis, prostate cancer survivors who reported sufficient discussion had CF scores that were, on average, 10 points higher than those who did not (95% CI: 0.3 to 19.7; *p* = 0.04).

### 3.6. Nutritional Problems and Quality of Life

Table 6 shows that, in the combined sample across all three cancer types, reported sufficient discussions about nutrition-related problems were associated with higher QL scores (β = 5.7 points; 95% CI: 0.3 to 11.1; *p* = 0.04) in a fully adjusted model (N = 387). In the model without adjusting for cancer stage at diagnosis (N = 480), participants who reported sufficient discussions had QL scores that were, on average, 7.8 points higher than those who did not (95% CI: 3.0 to 12.6; *p* = 0.001).

In analyses stratified by cancer type, a statistically significant association with QL was observed among prostate cancer survivors (β = 12.4; 95% CI: 2.5 to 22.2; *p* = 0.02) and breast cancer survivors (β = 8.4; 95% CI: 1.0 to 15.7; *p* = 0.03). In the colorectal cancer subgroups, the estimates for QL were not statistically significant.

For PF and CF, estimates were generally imprecise across cancer strata. No statistically significant associations were observed in the combined sample between guidance on nutrition-related problems and PF (β = +2.3; 95% CI: −3.4 to +8.0; *p* = 0.42) or CF (β = +1.9; 95% CI: −6.0 to +2.2; *p* = 0.53). Similarly, no statistically significant associations were observed in the cancer-specific subgroup analyses, as shown in Table 6.

## 4. Discussion

The findings of the present study suggest that receiving sufficient nutrition-related guidance and discussions during cancer treatment may be associated with higher post-treatment quality of life, with the clearest association observed among participants with prostate cancer, while a similar but less consistent pattern was observed among women with breast cancer among adults diagnosed with cancer in Iceland.

Women with breast cancer were the youngest group on average and reported the lowest mean QL score at study entry. Sufficient nutritional guidance was reported most often by participants with colorectal cancer, who had the highest proportion of advanced-stage disease and reported more pronounced gastrointestinal symptoms when assessing their quality of life, on average, almost three years after diagnosis. In contrast, men with prostate cancer had the lowest treatment burden and were least likely to report receiving sufficient guidance.

Taken together, these findings suggest that sufficient nutritional guidance may have varied according to cancer type, treatment burden, and disease severity. This is consistent with previous studies showing that individualized nutritional counseling during cancer treatment can improve nutritional intake, nutritional status, and quality of life [16,24]. More recent evidence also supports the role of nutritional and dietary interventions in managing chemotherapy-induced gastrointestinal toxicity and supporting treatment tolerance [15]. In addition, an Icelandic study of older patients with various diseases suggests that specialized nutritional counseling may play an important role in preserving cognitive function [11]. Such counseling may also help address metabolic disturbances that can negatively affect the physical well-being of patients with cancer [3].

### 4.1. Patterns in the Provision of Nutritional Guidance

Participants with breast cancer reported a higher symptom burden during the past seven days than the other groups, with mean nausea and vomiting scores ranging from 3.08 to 5.56. This may suggest that greater treatment burden contributed to more frequent nutritional counseling. However, breast cancer survivors also represented the largest absolute number of stage I–II participants who did not discuss nutrition-related problems, suggesting a possible gap in proactive nutritional guidance among women with less advanced disease. Future studies should examine whether nutritional guidance has greater potential to support long-term QL when provided earlier in the disease trajectory.

In the prostate cancer group, 65% reported discussing dietary habits and 48% discussed nutrition-related problems, the lowest proportions across the cancer groups. This group also reported the lowest symptom burden during the past seven days, with mean scores for nausea, vomiting, and appetite loss consistently below 3.0. Nutritional discussions appeared more common among participants with advanced-stage disease; among those who discussed nutrition-related problems, 33% had stage III or IV disease. This pattern may suggest that nutritional guidance in prostate cancer is more often provided in response to more advanced or symptomatic disease, rather than routinely at earlier stages.

In the colorectal cancer group, reported discussions were most common, with 85% discussing dietary habits and 72% discussing nutrition-related problems. Discussions appeared more frequent among participants with stage III or IV disease, who represented 56% of those discussing dietary habits and 58% of those discussing nutrition-related problems. This group also had the largest proportion of stage III or IV cases overall. Mean nausea and vomiting scores during the past seven days were low, all below 2.5, whereas appetite loss scores were more variable, ranging from 2.0 to 5.4.

Across cancer types, participants who reported discussions about either dietary habits or nutrition-related problems were generally younger than those who did not report such discussions. This pattern may suggest that nutritional guidance during cancer treatment in Iceland is not provided uniformly across age groups and may be influenced by clinical severity. Older patients may face barriers to accessing nutritional counseling or may be less often offered such support. Alternatively, care for older patients may focus more on other clinical needs. This is important, as older adults are more likely to have comorbidities, and cancer patients with comorbidities generally have higher mortality rates than those without comorbidities [17].

ESPEN guidelines recommend that all patients with cancer be screened for malnutrition and receive appropriate nutritional treatment when indicated, regardless of age, sex, cancer type, or disease stage [3]. However, previous results from the same study population showed that only 19% of participants reported having been evaluated for the need for nutritional counseling, suggesting that systematic nutritional screening was not implemented to a great extent in Iceland in the years between 2015 and 2019 [7]. This proportion is considerably lower than in the present analysis, where 71% reported discussions about dietary habits and 57% reported discussions about nutrition-related problems with healthcare professionals. The difference likely reflects differences in measurement: the previous estimate referred specifically to evaluation for nutritional counseling, whereas the present study captured broader self-reported discussions with healthcare staff. Therefore, many of the reported discussions may have taken place with healthcare professionals other than dietitians. Nevertheless, the present findings suggest that nutritional guidance may vary across cancer types and patient characteristics.

### 4.2. Strengths and Limitations

This population-based study used high-quality data from the Icelandic Cancer Registry [25]. Strengths of this study include its relatively sizable sample, the inclusion of participants with different cancer types, and the availability of registry-based clinical information, which enabled comparisons across demographic factors and disease characteristics. Another strength is the assessment of several aspects of quality of life, including physical and cognitive functioning, which provides a more nuanced understanding of how nutrition-related guidance may be associated with post-treatment well-being.

Several limitations should be considered. Information was not available on the duration, content, provider, or quality of the dietary guidance, which limits the ability to determine which aspects of the guidance may have been most relevant to quality of life. Receipt of sufficient guidance was retrospectively self-reported approximately three years after diagnosis and may therefore have been affected by recall or reporting bias. Participants with greater health awareness or better current quality of life may have been more likely to recall previous guidance as sufficient which could have overestimated the association between reported nutrition-related discussions and quality of life. Conversely, participants with poorer quality of life or ongoing symptoms may have been more likely to remember unmet needs or insufficient guidance, which could have influenced the associations in the opposite direction. Assessment closer to the end of treatment might have provided a different understanding of the association between nutrition-related guidance and post-treatment outcomes.

Moreover, nutritional guidance was not randomly provided and may have varied according to cancer type, disease severity, treatment burden, or nutrition-related symptoms, resulting in potential confounding by indication. Although the analyses were adjusted for stage at diagnosis, number of treatments, sex, education, marital status, and comorbidities, residual and unmeasured confounding cannot be excluded. Nausea and appetite loss were assessed at the time of the survey and may not reflect symptoms experienced during treatment.

Because the study design was cross-sectional, the temporal direction of the associations cannot be established, and reverse causation cannot be excluded. The participation rate was 40%, and selection bias may have occurred if individuals with poorer health status, lower functioning, or less interest in lifestyle-related issues were less likely to participate. In addition, the relatively small sample size, particularly in the cancer-specific analyses, limited statistical precision. Given the number of statistical tests conducted, including analyses of two exposures, several quality-of-life outcomes, and cancer type-stratified analyses, the possibility of type I error cannot be excluded, particularly in subgroup analyses.

### 4.3. Future Research

More longitudinal cohort studies and randomized controlled trials are needed to determine whether specialized nutritional guidance during cancer treatment leads to sustained improvements in global quality of life, physical functioning, and cognitive functioning.

Future studies should also include objective nutritional and functional markers, such as body composition, handgrip strength, relevant biomarkers, and formal neurocognitive testing. This would improve understanding of post-treatment quality of life and the mechanisms that may influence recovery.

## 5. Conclusions

This study found modest but statistically significant associations between patient-reported sufficient discussions about dietary habits and/or nutrition-related problems during cancer treatment and higher post-treatment quality of life. No clear associations were observed with self-reported physical or cognitive functioning in these cross-sectional analyses.

Participants who were younger, had more advanced disease, or had received multiple treatments appeared more likely to report sufficient nutritional guidance. This may suggest that nutritional guidance is more often provided in response to perceived clinical need rather than as a routine preventive component of cancer care. In cancer type-stratified analyses, the clearest associations were observed among participants with prostate cancer, with statistically significant associations for both sufficient discussions about dietary habits and nutrition-related problems. Among women with breast cancer, the estimate for discussions about dietary habits suggested a positive association, while sufficient discussions about nutrition-related problems were statistically significantly associated with higher quality of life. In the colorectal cancer subgroup, no statistically significant associations were observed. This may reflect limited statistical power, residual confounding, or the influence of ongoing gastrointestinal symptoms, which may have affected quality of life independently of nutritional guidance.

Overall, these findings support stronger implementation of routine nutritional screening and early, needs-based dietetic assessment in oncology care, in line with ESPEN guidance [3]. Nutritional care should be made available to all patients as part of cancer treatment, rather than primarily being provided once nutritional problems or higher treatment burden have emerged.

## Figures and Tables

**Table 1:** Exposure description.

Variable	Questions	Response Options
Have discussed general dietary habits	Hospital healthcare professionals have discussed general dietary habits with me	Yes, sufficiently Yes, to some extent Slightly No, to no extent I can’t remember Not applicable
Have discussed nutritional problems	Hospital healthcare professionals have discussed my nutritional problems with me	Yes, sufficiently Yes, to some extent Slightly No, to no extent I can’t remember Not applicable

**Table 2:** Descriptive characteristics of participants who had completed breast cancer treatment.

Variables	Have Discussed Dietary Habits			Have Discussed Nutritional Problems		
	No	Yes, to Some Extent/Slightly	Yes, Sufficiently	No	Yes, to Some Extent/Slightly	Yes, Sufficiently
	n = 110	n = 152	n = 79	n = 134	n = 112	n = 54
Age at diagnosis, mean (SD)	58.4 (10.1)	55.4 (11.0)	56.8 (10.4)	59.0 (10.60)	54.8 (10.4)	54.7 (10.3)
Age at study entry, mean (SD)	61.5 (10.4)	58.4 (11.2)	59.7 (10.2)	61.9 (10.9)	57.9 (10.5)	58.0 (10.3)
Age distribution, n (%)						
18–49	15 (14.2)	25 (16.8)	13 (16.7)	17 (13.0)	18 (16.4)	12 (22.2)
50–69	66 (62.3)	98 (65.8)	51 (65.4)	80 (61.1)	75 (68.2)	36 (66.7)
70+	25 (23.6)	26 (17.4)	14 (17.9)	34 (26.0)	17 (15.5)	6 (11.1)
Height cm, mean (SD)	167.3 (5.3)	167.2 (5.9)	166.6 (6.2)	167.5 (6.0)	167.1 (5.8)	166.7 (6.5)
Weight kg, mean (SD)	77.7 (13.8)	77.2 (14.4)	78.7 (14.7)	78.6 (14.36)	76.5 (13.9)	79.0 (14.3)
BMI, medium (SD)	27.7 (4.7)	27.6 (4.9)	28.5 (5.6)	27.9 (4.6)	27.4 (4.8)	28.6 (5.5)
Education, n (%)						
Primary education	21 (20.8)	27 (18.2)	21 (29.2)	23 (18.5)	24 (22.2)	13 (26.0)
Upper secondary school	26 (25.7)	42 (28.4)	16 (22.2)	33 (26.6)	31 (28.7)	14 (28.0)
Trade school	10 (9.9)	17 (11.5)	12 (16.7)	16 (12.9)	12 (11.1)	5 (10.0)
University degree	44 (43.6)	62 (41.9)	23 (31.9)	52 (41.9)	41 (38.0)	18 (36.0)
Marital status, n (%)						
Married or in a relationship	77 (72.0)	119 (79.9)	54 (68.4)	96 (72.7)	85 (78.0)	39 (72.2)
Single or Widowed	30 (28.0)	30 (20.1)	25 (31.6)	36 (27.3)	24 (22.0)	15 (27.8)
Children under 18, n (%)						
None	92 (86.0)	116 (77.3)	60 (75.9)	112 (84.8)	83 (75.5)	38 (70.4)
One or more	15 (14.0)	34 (22.7)	19 (24.1)	20 (15.2)	27 (24.5)	16 (29.6)
Smoking, n (%)						
No	42 (38.5)	62 (41.3)	32 (40.5)	48 (36.4)	49 (44.1)	23 (42.6)
Former smoker	61 (56.0)	75 (50.0)	44 (55.7)	72 (54.5)	54 (48.6)	29 (53.7)
Yes	6 (5.5)	13 (8.7)	3 (3.8)	12 (9.1)	8 (7.2)	2 (3.7)
Alcohol consumption, n (%)						
0–3 times a month	78 (70.9)	120 (80.0)	54 (68.4)	95 (70.9)	87 (78.4)	42 (77.8)
1–2 times a week	22 (20.0)	26 (17.3)	20 (25.3)	26 (19.4)	23 (20.7)	9 (16.7)
3 times or more a week	10 (9.1)	4 (2.7)	5 (6.3)	13 (9.7)	1 (0.9)	3 (5.6)
Number of treatments, n (%)						
None or one	6 (5.5)	5 (3.3)	4 (5.1)	7 (5.2)	5 (4.5)	2 (3.7)
Two or more	104 (94.5)	147 (96.7)	75 (94.9)	127 (94.8)	107 (95.5)	52 (96.3)
EORTC QLQ C30						
Nausea and vomiting, mean (SD)	3.1 (7.8)	6.1 (13.2)	4.3 (11.6)	3.7 (8.8)	6.2 (13.8)	4.3 (12.1)
Appetite, mean (SD)	6.8 (16.7)	9.0 (20.8)	8.0 (16.4)	5.9 (15.5)	11.6 (23.3)	6.7 (15.1)
Constipation, mean (SD)	22.0 (27.5)	14.5 (24.2)	16.2 (25.9)	21.1 (27.1)	15.2 (24.3)	14.0 (23.4)
Diarrhea, mean (SD)	11.0 (22.3)	10.0 (18.2)	6.8 (17.7)	10.5 (21.6)	10.6 (18.8)	7.3 (18.2)
Quality of life, poor, n (%)	32 (29.6)	38 (25.2)	12 (15.2)	37 (28.0)	34 (30.6)	6 (11.1)
Physical function, poor, n (%)	32 (36.0)	49 (36.0)	20 (28.2)	37 (33.3)	38 (37.3)	14 (29.2)
Cognitive function, poor, n (%)	57 (59.4)	74 (53.2)	44 (62.0)	76 (65.0)	48 (46.6)	31 (62.0)
Cancer stage, n (%)						
1	45 (50.6)	62 (52.1)	25 (41.0)	58 (52.7)	38 (44.7)	15 (36.6)
2	36 (40.4)	43 (36.1)	19 (31.1)	38 (34.5)	33 (38.8)	17 (41.5)
3 or 4	8 (9.0)	14 (11.8)	17 (27.9)	14 (12.7)	14 (16.5)	9 (22.0)
Comorbidities, n (%)						
Yes	54 (49.1)	76 (50.0)	38 (48.1)	65 (48.5)	58 (51.8)	24 (44.4)

**Table 3:** Descriptive characteristics of participants who had completed prostate cancer treatment.

Variables	Have Discussed Dietary Habits			Have Discussed Nutritional Problems		
	No	Yes, to Some Extent/Slightly	Yes, Sufficiently	No	Yes, to Some Extent/Slightly	Yes, Sufficiently
	n = 48	n = 60	n = 29	n = 61	n = 37	n = 19
Age at diagnosis, mean (SD)	66.9 (7.1)	63.9 (7.1)	66.5 (7.4)	66.3 (7.4)	63.5 (5.7)	65.0 (7.8)
Age at study entry, mean (SD)	70.0 (7.1)	67.2 (6.9)	69.2 (7.8)	69.6 (7.2)	66.7 (5.3)	68.0 (8.8)
Age distribution, n (%)						
18–49	0 (0.0)	2 (3.4)	0 (0.0)	1 (1.7)	0 (0.0)	0 (0.0)
50–69	24 (51.1)	31 (53.4)	17 (58.6)	28 (46.7)	24 (66.7)	11 (57.9)
70+	23 (48.9)	25 (43.1)	12 (41.4)	31 (51.7)	12 (33.3)	8 (42.1)
Height cm, mean (SD)	180.5 (5.6)	180.2 (7.3)	179.6 (6.2)	180.1 (5.9)	180.7 (7.6)	179.5 (6.1)
Weight kg, mean (SD)	89.8 (15.8)	92.8 (14.8)	93.5 (11.1)	90.3 (15.9)	93.3 (13.7)	93.2 (11.9)
BMI, medium (SD)	27.5 (3.9)	28.8 (3.9)	29.0 (3.9)	27.8 (4.0)	28.8 (3.7)	28.9 (3.6)
Education, n (%)						
Primary education	1 (2.3)	6 (10.7)	4 (14.8)	3 (5.4)	3 (8.8)	3 (17.6)
Upper secondary school	8 (18.2)	6 (10.7)	4 (14.8)	8 (14.3)	3 (8.8)	1 (5.9)
Trade school	20 (45.5)	25 (44.6)	13 (48.1)	27 (48.2)	15 (44.1)	11 (64.7)
University degree	15 (34.1)	19 (33.9)	6 (22.2)	18 (32.1)	13 (38.2)	2 (11.8)
Marital status, n (%)						
Married or in a relationship	40 (87.0)	50 (86.2)	26 (89.7)	53 (88.3)	29 (82.9)	17 (89.5)
Single or Widowed	6 (13.0)	8 (13.8)	3 (10.3)	7 (11.7)	6 (17.1)	2 (10.5)
Children under 18, n (%)						
None	45 (95.7)	53 (89.8)	27 (93.1)	57 (95.0)	34 (94.4)	17 (89.5)
One or more	2 (4.3)	6 (10.2)	2 (6.9)	3 (5.0)	2 (5.6)	2 (10.5)
Smoking, n (%)						
No	21 (43.8)	22 (36.7)	10 (34.5)	27 (44.3)	14 (37.8)	8 (42.1)
Former smoker	25 (52.1)	31 (51.7)	17 (58.6)	30 (49.2)	18 (48.6)	9 (47.4)
Yes	2 (4.2)	7 (11.7)	2 (6.9)	4 (6.6)	5 (13.5)	2 (10.5)
Alcohol consumption, n (%)						
0–3 times a month	27 (56.2)	39 (65.0)	19 (65.5)	37 (60.7)	23 (62.2)	15 (78.9)
1–2 times a week	19 (39.6)	16 (26.7)	10 (34.5)	22 (36.1)	13 (35.1)	4 (21.1)
3 times or more a week	2 (4.2)	5 (8.3)	0 (0.0)	2 (3.3)	1 (2.7)	0 (0.0)
Number of treatments, n (%)						
None or one	28 (58.3)	37 (61.7)	17 (58.6)	40 (65.6)	19 (51.4)	13 (68.4)
Two or more	20 (41.7)	23 (38.3)	12 (41.4)	21 (34.4)	18 (48.6)	6 (31.6)
EORTC QLQ C30						
Nausea and vomiting, mean (SD)	0.8 (3.6)	1.1 (4.2)	0.0 (0.0)	1.0 (3.9)	1.3 (4.5)	0.0 (0.0)
Appetite, mean (SD)	0.8 (5.2)	3.6 (10.4)	0.0 (0.0)	2.5 (8.9)	2.5 (8.9)	0.0 (0.0)
Constipation, mean (SD)	9.8 (17.1)	8.7 (16.9)	3.0 (9.8)	7.1 (15.3)	11.1 (18.5)	4.8 (12.1)
Diarrhea, mean (SD)	5.0 (12.1)	11.8 (21.9)	4.6 (11.7)	5.8 (14.3)	14.4 (24.3)	2.4 (8.9)
Quality of life, poor, n (%)	7 (14.9)	10 (16.9)	2 (6.9)	9 (15.0)	6 (16.7)	2 (10.5)
Physical function, poor, n (%)	8 (19.5)	11 (21.6)	4 (20.0)	10 (19.2)	8 (25.0)	3 (25.0)
Cognitive function, poor, n (%)	8 (19.0)	10 (20.8)	3 (13.0)	10 (18.9)	7 (25.0)	2 (13.3)
Cancer stage, n (%)						
1	25 (58.1)	21 (47.7)	8 (36.4)	30 (57.7)	9 (36.0)	6 (40.0)
2	10 (23.3)	10 (22.7)	7 (31.8)	11 (21.2)	6 (24.0)	6 (40.0)
3 or 4	8 (18.6)	13 (29.5)	7 (31.8)	11 (21.2)	10 (40.0)	3 (20.0)
Comorbidities, n (%)						
Yes	26 (54.2)	31 (51.7)	14 (48.3)	33 (54.1)	18 (48.6)	9 (47.4)

**Table 4:** Descriptive characteristics of participants who had completed colorectal cancer treatment.

Variables	Have Discussed Dietary Habits			Have Discussed Nutritional Problems		
	No	Yes, to Some Extent/Slightly	Yes, Sufficiently	No	Yes, to Some Extent/Slightly	Yes, Sufficiently
	n = 20	n = 62	n = 50	n = 32	n = 51	n = 30
Age at diagnosis, medium (SD)	66.1 (5.0)	61.2 (9.6)	62.7 (9.5)	63.7 (7.2)	60.8 (9.4)	63.3 (9.5)
Age at study entry, medium (SD)	68.6 (6.3)	64.4 (9.8)	65.5 (9.7)	66.4 (8.1)	63.7 (9.1)	66.2 (10.0)
Age distribution, n (%)						
18–49	0 (0.0)	3 (4.8)	2 (4.0)	0 (0.0)	4 (7.8)	1 (3.3)
50–69	12 (60.0)	40 (64.5)	33 (66.0)	21 (65.6)	35 (68.6)	21 (70.0)
70+	8 (40.0)	19 (30.6)	15 (30.0)	11 (34.4)	12 (23.5)	8 (26.7)
Height cm, medium (SD)	170.9 (8.6)	174.7 (8.4)	176.3 (9.9)	173.7 (7.8)	173.8 (9.8)	176.6 (9.5)
Weight kg, medium (SD)	88.0 (20.8)	84.4 (14.0)	90.5 (20.8)	86.7 (19.6)	84.4 (14.9)	91.9 (19.3)
BMI, medium (SD)	30.1 (6.4)	27.6 (4.1)	28.9 (5.1)	28.6 (5.7)	28.0 (4.5)	29.3 (4.9)
Education, n (%)						
Primary education	5 (27.8)	10 (17.9)	5 (11.4)	8 (25.0)	8 (17.4)	4 (15.4)
Upper secondary school	2 (11.1)	17 (30.4)	13 (29.5)	3 (9.4)	14 (30.4)	8 (30.8)
Trade school	5 (27.8)	9 (16.1)	8 (18.2)	9 (28.1)	7 (15.2)	4 (15.4)
University degree	6 (33.3)	20 (35.7)	18 (40.9)	12 (37.5)	17 (37.0)	10 (38.5)
Marital status, n (%)						
Married or in a relationship	14 (70.0)	50 (80.6)	42 (85.7)	24 (75.0)	42 (82.4)	27 (90.0)
Single or Widowed	6 (30.0)	12 (19.4)	7 (14.3)	8 (25.0)	9 (17.6)	3 (10.0)
Children under 18, n (%)						
None	19 (95.0)	54 (87.1)	45 (90.0)	30 (93.8)	44 (86.3)	26 (86.7)
One or more	1 (5.0)	8 (12.9)	5 (10.0)	2 (6.2)	7 (13.7)	4 (13.3)
Smoking, n (%)						
No	6 (30.0)	19 (30.6)	17 (34.0)	8 (25.0)	17 (33.3)	8 (26.7)
Former smoker	14 (70.0)	38 (61.3)	27 (54.0)	24 (75.0)	29 (56.9)	17 (56.7)
Yes	0 (0.0)	5 (8.1)	6 (12.0)	0 (0.0)	5 (9.8)	5 (16.7)
Alcohol consumption, n (%)						
0–3 times a month	12 (60.0)	41 (66.1)	32 (64.0)	18 (56.2)	35 (68.6)	17 (56.7)
1–2 times a week	5 (25.0)	15 (24.2)	15 (30.0)	8 (25.0)	13 (25.5)	11 (36.7)
3 times or more a week	3 (15.0)	6 (9.7)	3 (6.0)	6 (18.8)	3 (5.9)	2 (6.7)
Number of treatments, n (%)						
None or one	8 (40.0)	24 (38.7)	23 (46.0)	14 (43.8)	19 (37.3)	12 (40.0)
Two or more	12 (60.0)	38 (61.3)	27 (54.0)	18 (56.2)	32 (62.7)	18 (60.0)
EORTC QLQ C30						
Nausea and vomiting, medium (SD)	1.0 (4.2)	3.0 (7.9)	0.0 (0.0)	2.4 (7.5)	2.6 (7.1)	0.0 (0.0)
Appetite, medium (SD)	2.0 (8.1)	5.6 (12.5)	5.0 (17.8)	4.6 (11.7)	5.3 (12.3)	5.8 (21.7)
Constipation, medium (SD)	14.6 (29.7)	16.4 (23.9)	11.7 (22.1)	16.7 (28.0)	16.3 (24.2)	13.0 (24.1)
Diarrhea, medium (SD)	21.6 (33.2)	21.8 (25.8)	13.7 (19.8)	24.4 (33.8)	22.7 (23.6)	12.1 (16.4)
Quality of life, poor, n (%)	1 (5.0)	10 (16.1)	9 (18.0)	5 (15.6)	3 (5.9)	7 (23.3)
Physical function, poor, n (%)	10 (58.8)	19 (35.2)	13 (32.5)	13 (48.1)	13 (29.5)	13 (52.0)
Cognitive function, poor, n (%)	2 (12.5)	22 (38.6)	9 (22.5)	7 (24.1)	15 (33.3)	6 (26.1)
Cancer stage, n (%)						
1	8 (44.4)	8 (16.3)	12 (25.0)	9 (31.0)	5 (12.2)	8 (28.6)
2	2 (11.1)	19 (38.8)	7 (14.6)	7 (24.1)	15 (36.6)	1 (3.6)
3 or 4	8 (44.4)	22 (44.9)	29 (60.4)	13 (44.8)	21 (51.2)	19 (67.9)
Comorbidities, n (%)						
Yes	11 (55.0)	36 (58.1)	33 (66.0)	21 (65.6)	26 (51.0)	19 (63.3)

**Table 5:** Associations between reported sufficient discussions about general dietary habits and quality of life (QL), physical functioning (PF), and cognitive functioning (CF), overall and by cancer diagnosis.

Cancer Type	Outcome	β (Points) (Coefficient)	95% Cl (β)	*p*-Value	N
All cancers	QL	+5.6	+0.8 to +10.5	0.02	443
All cancers	PF	+4.1	−1.0 to +9.1	0.11	379
All cancers	CF	+3.0	−2.5 to +8.5	0.28	388
Breast	QL	+6.4	−0.3 to +13.2	0.06	244
Breast	PF	+6.2	−0.5 to +12.9	0.07	213
Breast	CF	+1.4	−7.0 to +9.8	0.75	219
Prostate	QL	+12.4	+2.5 to +22.2	0.01	98
Prostate	PF	+2.2	−7.4 to +11.7	0.65	79
Prostate	CF	+6.1	−2.5 to +14.6	0.16	80
Colorectal	QL	+3.4	−10.3 to +17.0	0.63	101
Colorectal	PF	+10.7	−5.5 to +27.0	0.19	87
Colorectal	CF	−0.7	−14.4 to +13.0	0.92	89

Adjusted for age, marital status, education, number of cancer treatments, stage at diagnosis, body mass index, tobacco- and alcohol use, and comorbidities.

**Table 6:** Associations between reported sufficient discussions about nutritional problems and quality of life (QL), physical functioning (PF), and cognitive functioning (CF) overall and by cancer diagnosis.

Cancer Type	Outcome	β (Points) (Coefficient)	95% Cl (β)	*p*-Value	N
All cancers	QL	+5.7	+0.3 to +11.1	0.04	387
All cancers	PF	+2.3	−3.4 to +8.0	0.42	330
All cancers	CF	+1.9	−6 to +2.2	0.53	339
Breast	QL	+8.4	+1.0 to +15.7	0.03	215
Breast	PF	+6.4	−1.1 to +13.8	0.09	187
Breast	CF	+0.5	−8.4 to +9.4	0.91	194
Prostate	QL	+14.0	+2.3 to +25.7	0.02	83
Prostate	PF	+0.8	−10.9 to +12.5	0.90	67
Prostate	CF	+7.8	−2.4 to +18.0	0.13	68
Colorectal	QL	−2.2	−15.3 to +11.0	0.60	89
Colorectal	PF	−4.1	−19.4 to +11.3	0.60	76
Colorectal	CF	−3.3	−12.8 to +6.3	0.50	72

Adjusted for age, marital status, education, number of cancer treatments, stage at diagnosis, body mass index, tobacco and alcohol use, and comorbidities.

## Data Availability

The data supporting the findings of this study are available from the Icelandic Compass study upon reasonable request. Access to the data is subject to approval by the study investigators and relevant ethical and data protection requirements, as the data contain information that could compromise participant privacy.
